# Markers of Tumor-Initiating Cells Predict Chemoresistance in Breast Cancer

**DOI:** 10.1371/journal.pone.0015630

**Published:** 2010-12-20

**Authors:** Chang Gong, Herui Yao, Qiang Liu, Jingqi Chen, Junwei Shi, Fengxi Su, Erwei Song

**Affiliations:** 1 Breast Tumor Center, Sun Yat-Sen Memorial Hospital, Sun Yat-Sen University, Guangzhou, People's Republic of China; 2 Department of Medical Oncology, Dana-Farber Cancer Institute, Boston, Massachusetts, United States of America; 3 School of Life Sciences, Sun Yat-Sen University, Guangzhou, People's Republic of China; Garvin Institute of Medical Research, Australia

## Abstract

**Purpose:**

Evidence is lacking whether the number of breast tumor-initiating cells (BT-ICs) directly correlates with the sensitivity of breast tumors to chemotherapy. Here, we evaluated the association between proportion of BT-ICs and chemoresistance of the tumors.

**Methods:**

Immunohistochemical staining(IHC) was used to examine the expression of aldehyde dehydrogenase 1 (ALDH1) and proliferating cell nuclear antigen, and TUNEL was used to detect the apoptosis index. The significance of various variables in patient survival was analyzed using a Cox proportional hazards model. The percentage of BT-ICs in breast cancer cell lines and primary breast tumors was determined by ALDH1 enzymatic assay, CD44^+^/CD24^−^ phenotype and mammosphere formation assay.

**Results:**

ALDH1 expression determined by IHC in primary breast cancers was associated with poor clinical response to neoadjuvant chemotherapy and reduced survival in breast cancer patients. Breast tumors that contained higher proportion of BT-ICs with CD44^+^/CD24^−^ phenotype, ALDH1 enzymatic activity and sphere forming capacity were more resistant to neoadjuvant chemotherapy. Chemoresistant cell lines AdrR/MCF-7 and SK-3rd, had increased number of cells with sphere forming capacity, CD44^+^/CD24^−^ phenotype and side-population. Regardless the proportion of T-ICs, FACS-sorted CD44^+^/CD24^−^ cells that derived from primary tumors or breast cancer lines were about 10–60 fold more resistant to chemotherapy relative to the non- CD44^+^/CD24^−^ cells and their parental cells. Furthermore, our data demonstrated that MDR1 (multidrug resistance 1) and ABCG2 (ATP-binding cassette sub-family G member 2) were upregulated in CD44^+^/CD24^−^ cells. Treatment with lapatinib or salinomycin reduced the proportion of BT-ICs by nearly 50 fold, and thus enhanced the sensitivity of breast cancer cells to chemotherapy by around 30 fold.

**Conclusions:**

These data suggest that the proportion of BT-ICs is associated with chemotherapeutic resistance of breast cancer. It highlights the importance of targeting T-ICs, rather than eliminating the bulk of rapidly dividing and terminally differentiated cells, in novel anti-cancer strategies.

## Introduction

Chemotherapy is an important component in the treatment paradigm for breast cancers. However, despite a rapid shrinkage in tumor mass following chemotherapeutic cycles, breast cancers may recur and develop distal metastasis later on. We now know that molecular mechanisms responsible for chemotherapeutic resistance of breast cancers are rather complicated, which involve overexpression of ATP-binding cassette transporters, anti-apoptotic factors [1] [2] and kinases for DNA repairing [3], [4]. Targeting any single molecule is not sufficient to reverse chemotherapeutic resistance [5], suggesting that multiple molecular pathways may contribute to the sensitivity of breast cancer cells to chemotherapy. Therefore, it is more important to identify and eliminate the subpopulation of cancer cells that are refractory to chemotherapeutic drugs.

Recently, accumulating evidence demonstrates that a wide variety of malignant tumors may be driven by a small subset of “tumor-initiating cells (T-ICs)” [6], [7] that display similar biological features of normal stem cells. Breast tumor-initiating cells (BT-ICs) form spherical clusters (“mammosphere”) in suspension cultures due to their self-renewal capacity [8]. BT-ICs also overexpress aldehyde dehydrogenase 1 (ALDH1) and bear the phenotype of CD44^+^/CD24^−^ [7], [9]. It has been shown that BT-ICs are more resistant to chemotherapy than non-BT-ICs due to multiple molecular mechanisms [10], including overexpression of ATP-binding cassette transporters [11] and enhanced ability of surviving [12], [13] and DNA damage repairing [12], [13]. Furthermore, self-renewing T-ICs can be selectively enriched or induced by chemotherapy [11]. However, whether the percentage of T-ICs within human tumors predict chemoresistance remains unknown [14].Therefore, we hypothesize that the proportion of BT-ICs may correlate with chemotherapeutic sensitivity of breast cancers, and reducing BT-ICs may reverse chemoresistance in the malignancy.

In our present study, we found that ALDH1 expression was associated with chemotherapeutic efficacy and clinical outcome of breast cancer patients, Furthermore, breast cancers containing a higher proportion of BT-ICs were more resistant to chemotherapy. BT-ICs isolated from various primary tumors or cell lines are equally resistant to chemotherapeutic drugs. In addition, reducing the number of breast T-ICs with Lapatinib and salinomycin sensitized BT-ICs to chemotherapy. These data suggest that the proportion of BT-ICs contributes to chemotherapeutic resistance of breast cancer.

## Results

### ALDH1 expression correlates with clinical outcome of breast cancer patients

**Figure 1:**
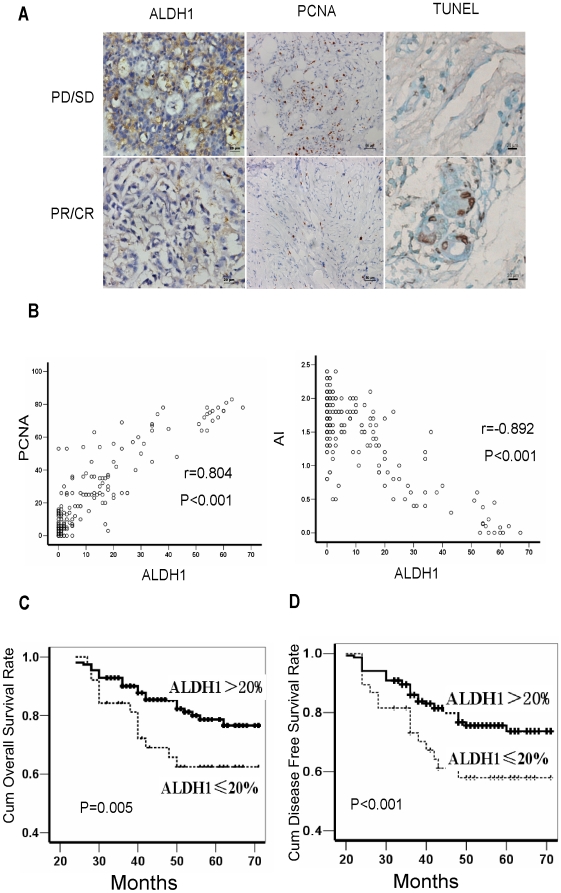
Aldehyde dehydrogenase (ALDH1) expression correlates with clinical outcome of breast cancer patients. (A) Immunohistochemical staining shows tumors with poor clinical response (progressive or stable disease, PD/SD) to neo-adjuvant chemotherapy express high ALDH1 (>20% positive cancer cells) in pre-chemotherapy samples, and tumors with partal response (PR) express low ALDH1 (≤20% positive cancer cells). High proliferating cell nuclear antigen (PCNA)(>25% positive cancer cells) and poor apoptosis are observed in tumors with PD/SD after neo-adjuvant chemotherapy. Representative images of ALDH1 (×200), PCNA (×200) and Terminal deoxynucleotidyl transferase (TdT)-mediated dUTP labeling (TUNEL,×400). (B) Surgically removed tumor samples obtained after neoadjuvant chemotherapy from patients with low ALDH1 have higher percentage of TUNEL-staining cells (r = −0.892, p<0.001), but lower percentage of PCNA-staining cells (r = 0.804, p<0.001). (C–D)Kaplan-Meier curves with log rank tests show statistical difference in overall survival (Log Rank = 7.987, p = 0.005) (C) and disease-free survival (Log Rank = 19.347, P<0.001) (D) between patients with high ALDH1 expression and low ALDH1 expression.

ALDH1 serves as a specific marker for normal and malignant human mammary stem cells [9]. To determine whether the number of BT-ICs is associated with chemotherapeutic efficacy, we performed immunohistochemical staining to examine ALDH1 expression in 192 cases of invasive ductal carcinomas of the breast obtained by core-needle biopsy prior to pre-operative neoadjuvant chemotherapy, and evaluated the expression level following the criteria of a previous study [15]. Here, 38 out of the 192 cases (19.8%) of breast cancers were classified as high ALDH1 expression (>20% positive cancer cells) (Table 1). The treatment paradigms including endocrine therapy and chemotherapy were comparable in patients with high or low ALDH1 expression (Table S1). ALDH1 expression was significantly associated with clinical staging, differentiation, tumor size and lymph node metastasis of the patients(Figure 1A, Table 1 ). More importantly, according to the Response Evaluation Criteria in Solid Tumors (RECIST), the clinical response rate, including partial and complete remission (PR+CR) was higher in patients with low ALDH1 than those with high ALDH1 (81% vs. 53%, x^2^ = 15.926; p<0.001) (Figure S1). In addition, breast cancers with low ALDH1 expression in their biopsies obtained prior to chemotherapy had higher percentage of apoptotic cells and lower percentage of proliferating cells in the corresponding surgical specimens obtained after neoadjuvant chemotherapy (Figure 1B, p<0.001), suggesting that ALDH1 expression in breast cancers is reversely correlated with apoptosis and proliferation inhibition induced by chemotherapy. Moreover, patients with low ALDH1 had longer overall survival (OS) and disease free survival (DFS) as compared with those with high ALDH1. The cumulative overall survival rate at 50 months for patients with low ALDH1 was 82.3%, but was only 55.1% for those with high ALDH1 (Log Rank = 7.987, p = 0.005, Figure 1C, Table S2). Similarly, the cumulative disease free survival rate at 50 months for patients with low ALDH1 was 77.3%, significantly higher than that for those with high ALDH1 expression (42%, Log Rank = 19.347, p<0.001, Figure 1D,Table S2). We next performed a Cox multivariate analysis of DFS, taking account of tumor size, clinical staging, differentiation, lymph node metastasis, estrogen receptor(ER), human epidermal growth factor receptor 2(Her2), proliferating cell nuclear antigen(PCNA) status, apoptosis index (AI) and ALDH1 expression as categorical variables. In the final multivariate COX regression model, ALDH1 expression in breast cancer patients was associated with poor survival prognosis (hazard ratio = 2.197, p = 0.013) independent of other clinical covariates, suggesting that ALDH1 can be used as an independent prognostic factor for breast cancers (Table S3). Collectively, our data suggest that expression of ALDH1, a marker of BT-IC, may indicate chemotherapy resistance and poor clinical outcome in breast cancer patients.

**Table 1:** Correlation among clinicopathologic status and the expression of ALDH1, PCNA and apoptosis.

characteristics	Subgroup	ALDH1			PCNA			AI		
		≤20%	>20%	P	≤20%	>20%	P	No.	Mean±SD	P
Age	≤40 years	51	11	0.916	36	26	0.227	62	1.47±0.61	0.721
	>40 years	103	27		86	44		130	1.44±0.58	
ER	Positive	113	26	0.541	92	47	0.242	53	1.41±0.60	0.578
	Negative	41	12		30	23		139	1.46±0.59	
PR	Positive	96	22	0.668	77	41	0.359	118	1.48±0.57	0.540
	Negative	58	16		45	29		74	1.43±0.60	
HER2	Positive	21	7	0.304	14	14	0.102	28	1.45±0.59	0.727
	Negative	133	31		108	56		164	1.41±0.59	
Clinical Stage	II	114	6	<0.001	96	24	<0.001	120	1.50±0.52	0.116
	III	40	32		26	46		72	1.37±0.67	
Grade	I	15	1	<0.001	13	3	0.145	16	1.39±0.52	0.834
	II	86	32		64	45		109	1.44±0.59	
	III	14	53		45	22		67	1.48±0.61	
Tumor size	T1	31	0	<0.001	31	0	<0.001	31	1.65±0.40	<0.001
	T2	102	15		82	35		117	1.51±0.55	
	T3	21	23		9	35		44	1.14±0.70	
Node Metastasis	1–3	131	23	<0.001	106	48	0.013	154	1.52±0.51	0.030
	≥4	22	14		16	22		38	1.35±0.67	

ALDH1 = aldehyde dehydrogenase 1; AI = apoptotic index, percentage of apoptotic cancer cells.

### BT-IC proportion correlates with poor clinical response to neo-adjuvant chemotherapy

**Figure 2:**
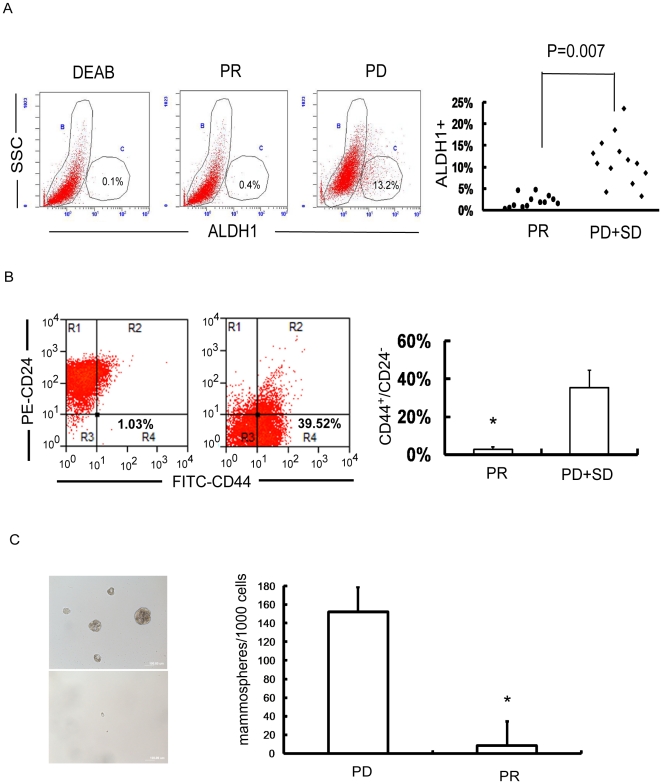
Breast tumor initiating cell (BT-IC) proportion correlates with poor clinical response to neo-adjuvant chemotherapy. (A) The example of ALDH1^+^ primary cancer cells in the progressive disease (PD) samples (symbol “•”, n = 13) is 13.2% whereas 0.4% in partial response (PR) samples (symbol “⧫”, n = 13) (p = 0.007). All these 26 cancer tissue samples were obtained before chemotherapy. (B) Breast tumors with progressive or stable disease(PD/SD) (7 cases) have more cancer cells CD44^+^/CD24^−^ phenotype than those with PR (7 cases) (35.0%±9.13% vs. 1.56%±0.33%, p<0.001). (C) Mammosphere formation of primary cancer cells in breast tumors with PD/SD is 10–20 fold higher than those with PR (15.2% vs. 0.83%, p<0.001).

In order to directly evaluate the contribution of BT-IC to chemotherapeutic response, we isolated primary cancer cells from the biopsies of 26 breast cancer patients obtained prior to chemotherapy and correlated ALDH1 enzymatic activity, CD44^+^/CD24^−^ phenotype and mammosphere forming rate with the efficacy of chemotherapy. Among all the 26 patients who received neoadjuvant chemotherapy of FEC (5-Fluorouracil 500mg/m^2^, Epirubucin 90mg/m^2^ and Cyclophosphamide 500mg/m^2^) regimen for 4 cycles, 13 patients had partial or complete response (PR/CR), while the other 13 patients had stable or progressive diseases (PD/SD) (Table S4). The percentage of ALDH1^+^ cells was significantly higher in breast cancers with PD/SD than those with PR/CR (11.54%±5.65% vs. 2.08%±1.42%, p = 0.007, Figure 2A). Similarly, breast cancers with PD/SD had higher percentage of CD44^+^/CD24^−^cancer cells than those with PR/CR (35.0%±9.13% vs. 1.56±0.33%, p<0.001, Figure 2B). Moreover, mammosphere formation of primary cancer cells in breast tumors with PD/SD was 10–20 fold higher than those with PR/CR (p<0.001, Figure 2C). These findings demonstrate that a higher proportion of T-ICs is associated with poor clinical response of primary breast cancers to chemotherapy, suggesting that the number of BT-ICs plays a pivotal role in chemotherapeutic resistance of breast cancers.

### BT-ICs from various primary tumors and cell lines are resistant to chemotherapy

**Figure 3:**
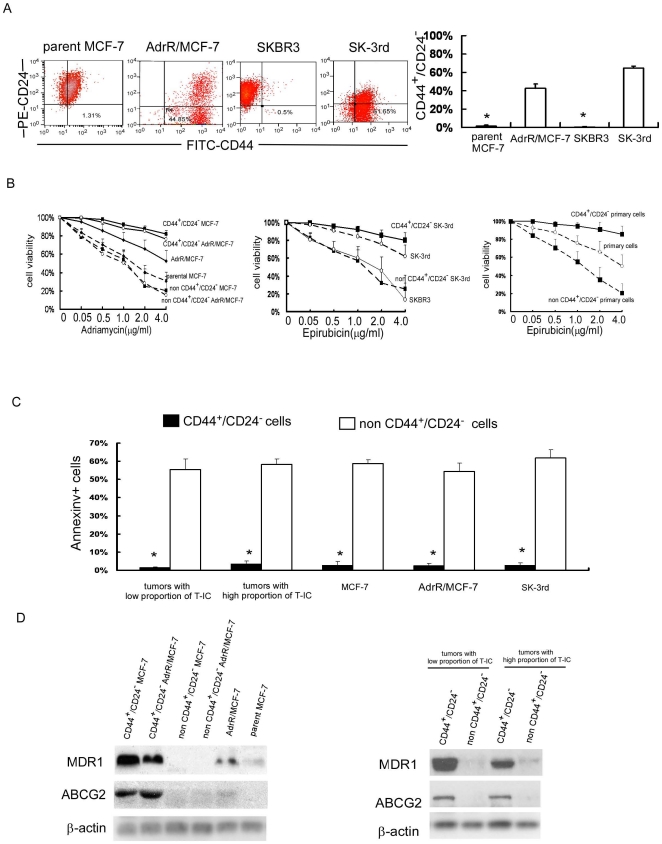
BT-ICs are intrinsic chemoresistance independent of individual tumors and cell lines. (A) The percentage of adriamycin resistant MCF-7 (AdrR/MCF-7) cells and epirubicin resistant SKBR3 (sk-3rd) cells with the phenotype of CD44^+^CD24^−^ is significantly higher than parental MCF-7 line and SKBR3 line tested by FACS (42.38±4.80% vs. 1.82%±0.64%, 64.34±2.62% vs. 0.68%±0.16%, p<0.001). (B) FACS-sorted CD44^+^CD24^−^ MCF-7 and CD44^+^CD24^−^ AdrR/MCF-7 was about 10–60 fold higher resistant to adriamycin relative to the parental cells and non-CD44^+^CD24^−^ cells. CD44^+^CD24^−^ SK-3rd and primary cells were about 25 fold higher resistant to epirubicin relative to parental cells and non-CD44^+^CD24^−^ cells by using cell viability assay. (C)Compared with non-BT-ICs, chemotherapy (adriamycin or epriubicin 0.5µg/ml for 24 hours) increased Annexin V positive cells of non-CD44^+^/CD24^−^cells, which derived from tumors with high or low proportion T-ICs, MCF-7, AdrR/MCF-7 and SK-3rd breast cancer cell lines containing different number of T-ICs (p<0.001). (D) MDR1 (multidrug resistance 1) and ABCG2(ATP-binding cassette sub-family G member 2) protein expression is both up-regulated in CD44^+^/CD24^−^ cells from different cell lines and primary cells. All the experiments were repeated 3 times independently.

To evaluate whether T-ICs isolated from the primary breast tumors of different individuals or from different breast cancer lines are resistant to chemotherapy, we sorted for putative T-ICs from both clinical specimens and cell lines and compared the chemotherapeutic sensitivity of T-ICs with non-T-ICs. First, we examined the proportion of BT-ICs in AdrR/MCF-7 and SK-3rd, the breast cancer lines induced to be resistant to adriamycin and epirubicin. As shown in Figure 3A, the percentage of AdrR/MCF-7 cells and SK-3rd cells with the phenotype of CD44^+^/CD24^−^ was significantly higher than the parental MCF-7 cell line and SKBR3 cell line (42.38±4.80% vs. 1.82%±0.64%, 64.34±2.62% vs. 0.68%±0.16%, p<0.001). In agreement, the percentage of mammospheres formed in AdrR/MCF-7 line and SK-3rd line was significantly higher than the parental MCF-7 line and SKBR3 line (5.12±0.22% vs. 1.38±0.49%, 13.6±1.02% vs. 0.22±0.13% , p<0.001, Figure S2A and S2B). Next we investigated whether the chemotherapeutic sensitivity of T-ICs differ among individual tumors. Cell Counting Kit-8 was used to determine the number of viable cells upon treatment with adriamycin at increasing concentrations. As shown in Figure 3B, FACS-sorted CD44^+^/CD24^−^ cells have a prominent survival advantage over non-CD44^+^/CD24^−^ cells and their parental cells. Upon adriamycin treatment, the survival of CD44^+^/CD24^−^ MCF-7 and AdrR/MCF-7 were similar and were about 10–60 fold higher than those of non-CD44^+^/CD24^−^ cells and their parental cells. Likewise, CD44^+^/CD24^−^ SK-3rd were about 25-fold more resistant to epirubicin than the non-CD44^+^/CD24^−^ SK-3rd and the parental SKBR3 cells. CD44^+^/CD24^−^ primary cells were about 25-fold more resistant to epirubicin than the non-CD44^+^/CD24^−^ cells. In the absence of chemotherapy, survival was comparable in the cells with or without CD44^+^/CD24^−^ phenotype. Furthermore, regardless the proportion of T-ICs in the primary tumors or the breast cancer lines, adriamycin or epriubicin significantly increased the apoptosis of non-CD44^+^/CD24^−^cells but not that of CD44^+^/CD24^−^ cells determined by Annexin V staining (Figure 3C). Moreover, our data demonstrated that MDR1 and ABCG2 expression were both upregulated in CD44^+^/CD24^−^ cells from various cell lines and primary tumors (Figure 3D, Table S5), along with increased side-population (SP) (Figure S3). These results indicate that BT-ICs are the subpopulation of cancer cells in both the primary tumors and various cell lines that are resistant to chemotherapy and thus may determine the chemotherapeutic sensitivity of breast cancer.

### Reducing the proportion of BT-ICs sensitizes breast cancer cells to chemotherapy

**Figure 4:**
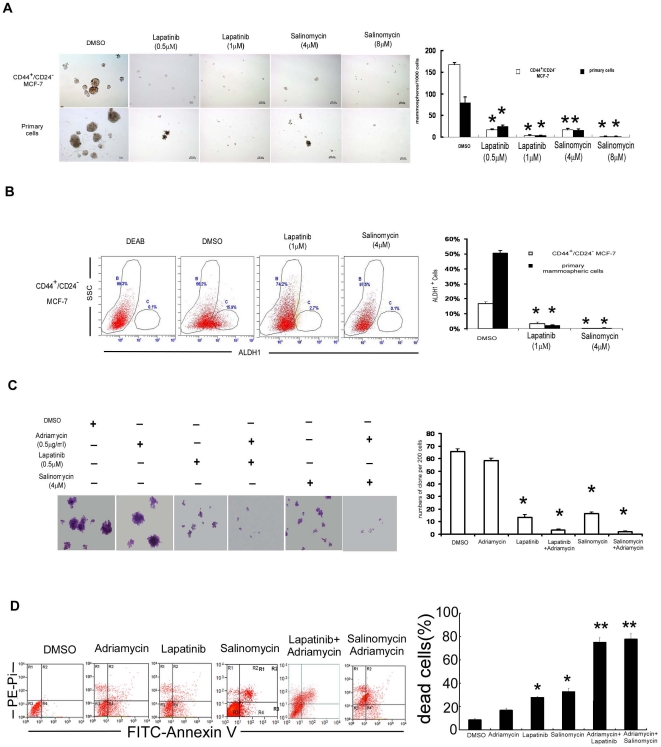
Reducing the proportion of BT-ICs sensitizes breast cancer cells to chemotherapy. (A)Treatment with salinomycin or lapatinib significantly reduced mammosphere formation in CD44^+^/CD24^−^ MCF-7 cells and primary cells by approximately 10 fold relative to DMSO control (p<0.001). (B) Treatment with salinomycin or lapatinib reduced the percentage of ALDH1^+^ in CD44^+^/CD24^−^ MCF-7 cells by 5–10 fold and primary cells by 50 fold. (C) Addition of lapatinib or salinomycin reduced the percentage of cloning efficiency of CD44^+^CD24^−^ MCF7 cells by approximately 10–30 fold (p<0.001). (D) The sensitivity of CD44^+^/CD24^−^ MCF-7 cells to adriamycin increased in the presence of lapatinib or salinomycin, as the dead cells increased from 27.8%±0.85% and 32.8%±2.6% to 75.1%±4.1%% and 77.9%±4.8% respectively determined by FACS analysis of Annexin V and PI staining(*p<0.01, **p<0.001, mean±SD). All the experiments were repeated 3 times independently.

Based on the above observations, we further investigated whether reducing the proportion of BT-ICs may sensitize breast cancers to conventional chemotherapy. Recent studies reported that lapatinib [16], a double EGFR/HER2 inhibitor, selectively killed the BT-ICs upon chemotherapy. In addition, salinomycin, an antimicrobial and anticoccidial agent, efficiently inhibits self-renewal of BT-ICs in breast cancer cell lines and reduces the expression of BT-IC specific genes in breast cancer tissues [17]. Here, CD44^+^/CD24^−^ BT-ICs sorted from MCF-7 line constitutively express EGFR, much higher than that in the parental MCF-7 and the AdrR/MCF-7 cells (Figure S4). More importantly, treatment with salinomycin or lapatinib significantly reduced mammosphere formation(p<0.001, Figure 4A and Figure S5) and the percentage of ALDH1^+^ cells by approximately 10–100 fold in CD44^+^/CD24^−^ BT-ICs isolated from MCF-7 cells, primary cells and SK-3rd cells (p<0.001, Figure 4B, Figure S6A and S6B). Although chemotherapy alone did not influence the cloning efficiency of CD44^+^CD24^−^ MCF-7 and SK-3rd cells, addition of lapatinib or salinomycin significantly reduced it by approximately 10–30 fold (p<0.001; Figure 4C, Figure S7). Moreover, combined treatment with adriamycin and lapatinib or salinomycin synergistically increased cell death from 27.8%±0.85% and 32.8%±2.6% to 75.1%±4.1%% and 77.9%±4.8% (Figure 4D), suggesting that reducing the proportion of BT-ICs enhances chemotherapeutic sensitivity in breast cancers.

## Discussion

It has been noted that tumor-initiating cells, or cancer stem cells, in solid malignancies are resistant to chemotherapy, but evidence is lacking whether the number of breast tumor-initiating cells directly correlates with chemotherapeutic sensitivity of the tumor. Here, we demonstrated that the percentage of ALDH1-positive cancer cells was associated with clinical chemotherapeutic response and prognosis in breast cancer patients. Breast tumors containing a higher proportion of BT-ICs were more resistant to chemotherapy. BT-ICs themselves were resistant to chemotherapy and their proportion in breast tumors determined chemotherapeutic sensitivity of the tumors. Furthermore, reducing the number of BT-ICs sensitized breast cancer cells to chemotherapy.

Growing evidence showed that the proportion of T-ICs varies among different primary tumors and different cancer cell lines [11], [12]. Breast cancer cells may develop biological features analogous to stem cells, especially when they undergo epithelial-mesenchymal transition (EMT) [18] or acquire amplification of human epidermal growth factor receptor 2 (HER2) gene [19]. Therefore, T-ICs are present in various proportions in primary breast tumors. Here, we employed specific markers to quantify the proportion of BT-ICs in primary breast cancers or breast cancer cell lines. Based on our data of flow cytometry, the CD44^+^/CD24^−^ and ALDH1^+^ primary breast cancer cells varied from 1.8% to 48% and 0.4% to 23.6% respectively, which was verified by ALDH1 immunohistochemical staining. This suggests that the proportion of T-ICs is different in individual breast tumors.

Chemotherapy has been shown to selectively enrich BT-ICs in the bulk of breast cancer cells. In contrast to non-BT-ICs that are probably not responsible for cancer recurrence or metastasis following chemotherapy [20], [21], growing evidences suggest that T-ICs are a subpopulation of cancer cells that drive tumor growth and recurrence. In our study, we analyzed the expression level of ALDH1 in primary breast cancers prior to pre-operative neoadjuvant chemotherapy and the breast cancer cells without drug exposure. These results indicated that poor clinical response and prognosis were associated with high proportion of BT-ICs that are resistant to chemotherapy. Similarly, compared with parental cells and non-CD44^+^/CD24^−^ cells, CD44^+^/CD24^−^ cells were 10–60 fold more resistant to chemotherapy. Taken together, these data suggest that the proportion of BT-ICs plays a key role in chemotherapeutic resistance and post-treatment recurrence of breast cancers, and thus contributes to the poor prognosis of breast cancer patients.

Chemotherapy is less efficient in BT-ICs due to a variety of mechanisms. In light of our results, compared with parental cells and drug resistant cells, CD44^+^/CD24^−^ cells are more likely to survive toxic insults probably due to up-regulated MDR1 and ABCG2. Like the normal tissue stem cells, multidrug resistance (MDR) transporters protect dormant cancer stem cells, and MDR is constitutively expressed at stable and high levels in T-ICs with stem-cell like features. Thus, BT-ICs are believed to be resistant to chemotherapy prior to receiving the treatment, which leads to their enrichment following chemotherapy. Several signaling pathways such as the epidermal growth factor receptor (EGFR), Hedgehog, Notch and Wnt signaling pathways [22] are involved in the regulation of treatment resistance in BT-ICs. Nevertheless, all these mechanisms have complicated cross link with one another [23]. Therefore, it is not sufficient to kill BT-ICs, especially in clinical therapy only by interfering with a single molecule.

The number of T-ICs is controlled by their own self-renewal, which is regulated by several signaling pathways such as the Hedgehog, Notch and Wnt pathways [22]. Inhibition of key regulatory pathways responsible for self-renewal could reduce the number of T-ICs. Recently, some studies showed that cancer stem cells can be targeted by lapatinib [16] and salinomycin [17]. We found that mammosphere formation rate and the percentage of cells with enzymatic activity of ALDH1 or CD44^+^/CD24^−^ profile in MCF-7 and SK-3rd cells decreased significantly in the presence of the lapatinib or salinomycin. Moreover, combined treatment with chemotherapy and lapatinib/salinomycin synergistically, rather than additively, increases breast cancer cell death, implying that targeting T-ICs may eradicate chemotherapeutic resistance and post-treatment recurrence in breast cancers. Furthermore, Lapatinib has been shown to sensitize cancer cells to chemotherapeutic drugs by inhibiting the EGFR, P-glycoprotein (Pgp) and breast cancer resistance protein (BCRP) [24].

The mechanisms of the drug resistance in T-ICs are very complicated. Our data suggest that the proportion of T-ICs directly correlates with chemotherapeutic resistance of breast cancer. Thus, it is important to quantify the proportion of BT-ICs to predict chemotherapeutic sensitivity. It also implies that anti-cancer therapy should shift from attempts to eliminate the bulk of rapidly dividing and terminally differentiated cells to targeting T-ICs within a tumor.

## Materials and Methods

### Ethics statement

At the time of initial diagnosis, all patients had provided consent in the sense that their tumor samples could be used for investigational purposes. Institutional approval from local research ethical committee was obtained for the conduct of the study (Internal Review and the Ethics Boards of the Sun Yat-Sen Memorial Hospital, Sun Yat-Sen University). Data were analyzed anonymously. Patients provided written consent so that their samples and clinical data could be used for investigational purposes.

### Patients and tumor specimens

Tumor tissues of primary invasive ductal carcinomas of the breast were obtained from 192 female patients with stage IIB and III prior to pre-operative neoadjuvant chemotherapy in the breast tumor center, Sun Yat-Sen Memorial Hospital, Sun Yat-Sen University from Feb 2003 to Dec 2008. All the patients underwent pre-operative neoadjuvant chemotherapy with 2 to 6 cycles of FEC regimen (5-Fluorouracil 500mg/m^2^, Epirubucin 90mg/m^2^ and Cyclophosphamide 500mg/m^2^). Subsequently, the patients underwent total mastectomy or breast conservation. The patients with breast conservation received postoperative radiotherapy delivered to the entire breast using photon beams (median dose 50Gy) and a boost to the primary tumor bed delivered by electron beams, for a total median dose of 66Gy. According to the clinical response of neoadjuvant chemotherapy determined by the Response Evaluation Criteria in Solid Tumors (RECIST), adjuvant FEC or Taxotere was selectively administered in the patients with PR/cCR or PD/SD. Endocrine therapy was administered for patients with positive hormonal receptor. Distal metastasis was not found in these patients upon diagnosis, but was identified in 54 cases during post-operative follow-up, overall survival (OS) and disease free survival (DFS) were analyzed as the clinical endpoints, the median follow-up time is 50 months.

Fresh tumor tissues were obtained from another 26 female breast cancer patients before any treatment via ultrasound-guided vacuum-assisted Vacora biopsy system (Bard Biopsy System, Tempe, AZ). The samples were received in the laboratory within 20 min and immediately mechanically disaggregated and digested with collagenase as described [7]. Single-cell suspensions were obtained by filtration through a 40µm filter.

### Immunohistochemistry

The level of ALDH1 and PCNA was tested by immunohistochemistry staining in paraffin-embedded tissue sections. Rabbit monoclonal ALDH1A1 antibody (ab52492, Abcam) (1∶100 dilutions) and anti-PCNA antibody (ab29, Abcam) (1∶6000 dilutions) were used as primary antibody. The rabbit IgG or mouse IgG was added as the negative control in each case. The proportion of positive tumor cells was evaluated by two pathologists who were blinded to the study endpoints and used to quantify the degree of the immunostaining. For the statistical analysis, we divided cases into two groups: low expression (ALDH1^+^≤20% or PCNA≤25%) and high expression (ALDH1^+^>20% or PCNA>25%) [9].

### Terminal deoxynucleotidyl transferase (TdT)–mediated dUTP labeling (TUNEL)

TUNEL assay was done using an In situ Apoptosis Detection Kit (R&D Systems). Briefly, after digesting with Protease K, TdT reaction mix was applied to the cells for incubation at 37°C for 60 min, followed by incubation with streptavidin horseradish peroxidase for 10 min. The final reaction of the product was visualized by 3,3′-diaminobenzidine. Approximately 1,000 tumor cells were counted (400×) in each section, and apoptotic index(AI) was expressed as the percentage of TUNEL-positive tumor cells.

### Cell culture and treatment

Cells (1000cells/ml) were cultured in suspension in serum-free DMEM-F12 (Gibco), supplemented with B27 (1∶50, Invitrogen), 20 ng/mL EGF (BD Biosciences), 0.4% bovine serum albumin (Sigma), and 4 mg/ml insulin (Sigma) to form mammospheres [25]. In all the mammosphere experiments, primary plating data are provided. Wild type (WT) MCF-7 human breast cancer cells were obtained from American Type Culture Collection (ATCC) and grown according to standard protocols. Adriamycin resistant MCF-7 (AdrR /MCF-7) cells were selected in stepwise increasing concentrations beginning at 10^−8^ M Adriamycin. When cells were able to survive at any given concentration of the drug, they were passaged in the concentrations which were 1.5-fold higher. Cells were subsequently obtained which were able to survive in 10^−5^ M adriamycin. Parental SKBR3 and epirubicin resistant SKBR3 (SK-3rd) cells were cultured in RPMI1640 containing 10%FBS [11]. Drug lapatinib (GW572016) and Salinomycin (Sigma, s4526) was dissolved in DMSO.

### Flow cytometry

Flow cytometric cell testing and sorting was performed on single-cell suspensions derived from cell lines and primary tumor specimens using an Epics Altra flow cytometer (Beckman Coulter). CD44 and CD24 expression was analyzed in cells following incubation in trypsin-EDTA or dissociation with a pipette and passage through a 40-µm sieve. At least 1×10^5^ cells were pelleted by centrifugation at 500 g for 5 min at 4°C, resuspended in 2µL of FITC-conjugated anti-CD44 (PharMingen) and 5µL of PE-conjugated anti-CD24 (PharMingen), then incubated at 4°C in the dark for 30 min. The labeled cells were washed twice and analyzed. The ALDEFLUOR kit (StemCell Technologies, Durham, NC, USA) was used to test ALDH enzymatic activity. As the negative control, for each sample of cells an aliquot was treated with 50 mmol/L diethylaminobenzaldehyde (DEAB), a specific ALDH inhibitor. SP analysis was performed as described [26]. In all the experiments, verapamil 50µmol/L was added with dye to confirm the SP. Dead cells were quantified using the Annexin V Apoptosis detection kit (Calbiochem, PF032).

### Western blotting

Protein extracts were resolved through SDS-PAGE, transferred to polyvinylidene difluoride membranes (Amersham Pharmacia Biotech), probed with ABCG2 antibody (1∶500,San Cruz, SC-18841) and Mdr-1 antibody (1∶200, San Cruz,SC-71557), then with HRP-conjugated anti-rabbit or anti-mouse IgG secondary antibody, and visualized by chemiluminescence.

### RT-PCR assays

MDR1 and EGFR mRNA expression were monitored by semi-quantitative RT-PCR. The sequence of the primers used for RT-PCR was as follow: MDR1 forward: 5′-AACGGAAGCCAGAACATTCC-3′; MDR1 reverse: 5′-AGGCTTCCTGTGGCAAAGAG-3′ [27]; EGFR forward: 5′-AGCCATGCCCGCATTAG CTC-3′; EGFR reverse: 5′-AAAGGAATGCAACTTCCCAA-3′ [28]. DNA products were run on 1.5% agarose gel and visualized by ethidium bromide using UV light. Data presented are representative of at least three independent experiments.

### Cell viability assay

Cells were seeded in a 96 well plate with 100uL media at a density of 1×10^4^ per well and incubate (37°C, 5% CO_2_) overnight to allow the adherent cells to attach to the wells. Adherent cells were cultured in media with 10% FBS, and mammospheric cells cultured in suspension media without FBS. Then the cultured cells were incubated (37°C, 5% CO_2_) with adriamycin for 24 hours. 20uL cell counting kit-8 solution (CCK-8,Alexis Biochemical Corp., San Diego, CA) was added to each well and incubated (37°C, 5% CO_2_) for 4 hours. Optical density was read at 560nm and subtracted background at 670nm.

### Statistical analysis

All statistical analyses were carried out using SPSS 13.0 (SPSS, Chicago, IL). Student's t-test and One-Way ANOVA were used to analyze the relationship between apoptotic index and clinicopathologic characteristics, and the difference between ALDH1, CD44^+^/CD24^−^ expression or mammosphere formation rate in the cell lines and clinical response. Chi-Square Test was applied to analyze the relationship between ALDH1,PCNA expression and clinicopathologic features. To measure the association between pairs of variables, Spearman order correlation were run. Survival curves were plotted by the Kaplan-Meier method and compared by the log-rank test. The significance of various variables for survival was analyzed by the Cox proportional hazards model in the multivariate analysis. All experiments for cell cultures were performed at least in triplicate. Results were expressed as mean ± SD. *P*<0.05 in all cases was considered statistically significant.

## Supporting Information

Figure S1 **The clinical response of neoadjuvant chemotherapy with ALDH1 expression.** According to Response Evaluation Criteria in Solid Tumors (RECIST), partial response (PR) ,clinical complete response (cCR), progressive or stable disease (PD/SD) were 16, 3, and 19 respectively among the patients with high ALDH1expression. In contrast, the patients with ALDH1 low expression were 123, 12 and 19 respectively. There was significant difference between the ALDH1 expression and clinical response (81% vs. 53%, x^2^ = 15.926; p<0.001).
(TIF)

Figure S2 **Mammospheres forming in chemotherapy sensitive and resistant breast cancer cells.** (A) Representative images of mammospheres for parental MCF-7, AdrR/MCF-7, CD44^+^/CD24^−^ MCF-7, SKBR3 and SK-3rd. (B) The percentage of mammospheres formed in CD44^+^/CD24^−^ MCF-7, AdrR/MCF-7 and sk-3rd cell lines was significantly higher than parental MCF-7 line and SKBR3 line (*p<0.001,mean±SD).
(TIF)

Figure S3 **High proportion of side-population (SP) in CD44^+^/CD24^−^ cells.** (A) Representative images of SP determined by FACS analysis. (B) Compared with non-CD44^+^CD24^−^ MCF-7, parental MCF-7 and AdrR/MCF-7, CD44^+^CD24^−^ MCF-7 was more sensitive to adriamycin (*p<0.01, **p<0.001, mean±SD). Results were from 3 independent experiments.
(TIF)

Figure S4 **Expression of EGFR in breast cancer cell lines.** In comparison to the parental MCF7 cells and AdrR/MCF7 cells, CD44^+^/CD24^−^ MCF-7 cells more constitutively expressed EGFR tested by RT-PCR. This experiment was repeated 3 times independently.
(TIF)

Figure S5 Salinomycin or lapatinib significantly reduced mammosphere formation of sk-3rd cells by approximately 10–100 fold relative to DMSO control (p<0.001). This experiment was repeated 3 times independently.
(TIF)

Figure S6 Treatment with salinomycin or lapatinib reduced the percentage of ALDH1^+^ in sk-3rd cells and primary cells by 10–50 folds.
(TIF)

Figure S7 Clone forming assay from 3 independent experiments showed that epirubicin combined with salinomycin and lapatinib reduced the cloning efficiency of SK-3rd cells compared with epirubicin alone (p<0.001).
(TIF)

Table S1 (DOC)

Table S2 (DOC)

Table S3 (DOC)

Table S4 (XLS)

Table S5 (XLS)
