# Supplementary figures and images for: Markers of Tumor-Initiating Cells Predict Chemoresistance in Breast Cancer
Source: PLoS One. 2010 Dec 20;5(12):e15630. doi: 10.1371/journal.pone.0015630 (PMC3004932; doi:10.1371/journal.pone.0015630)

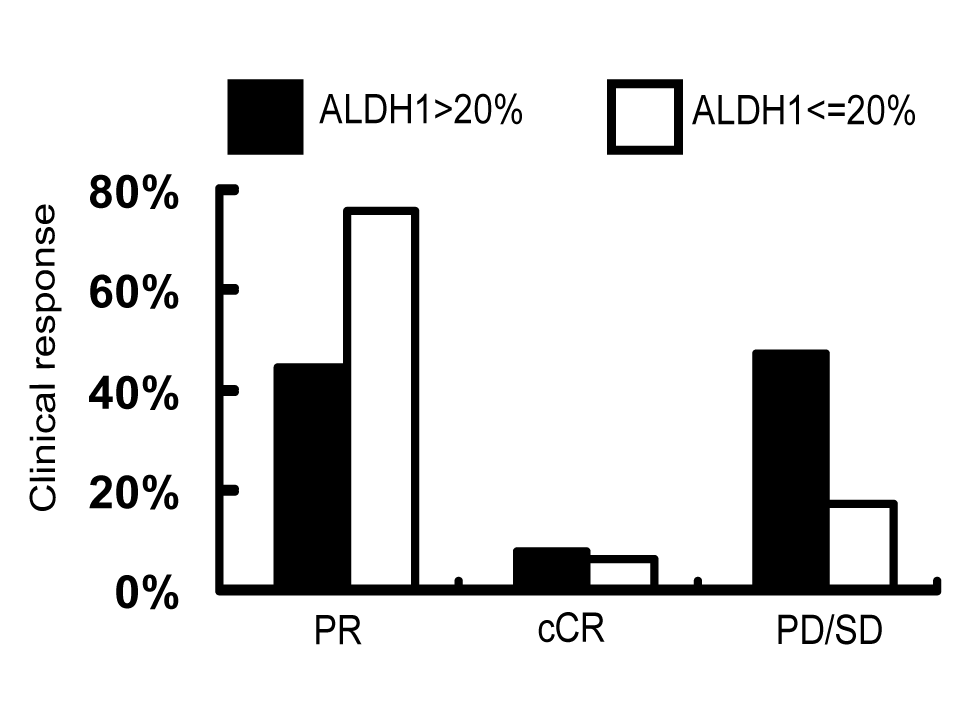

Supplement: Figure S1 — The clinical response of neoadjuvant chemotherapy with ALDH1 expression. According to Response Evaluation Criteria in Solid Tumors (RECIST), partial response (PR) ,clinical complete response (cCR), progressive or stable disease (PD/SD) were 16, 3, and 19 respectively among the patients with high ALDH1expression. In contrast, the patients with ALDH1 low expression were 123, 12 and 19 respectively. There was significant difference between the ALDH1 expression and clinical response (81% vs. 53%, x2 = 15.926; p<0.001). (TIF) [file pone.0015630.s001.tif]

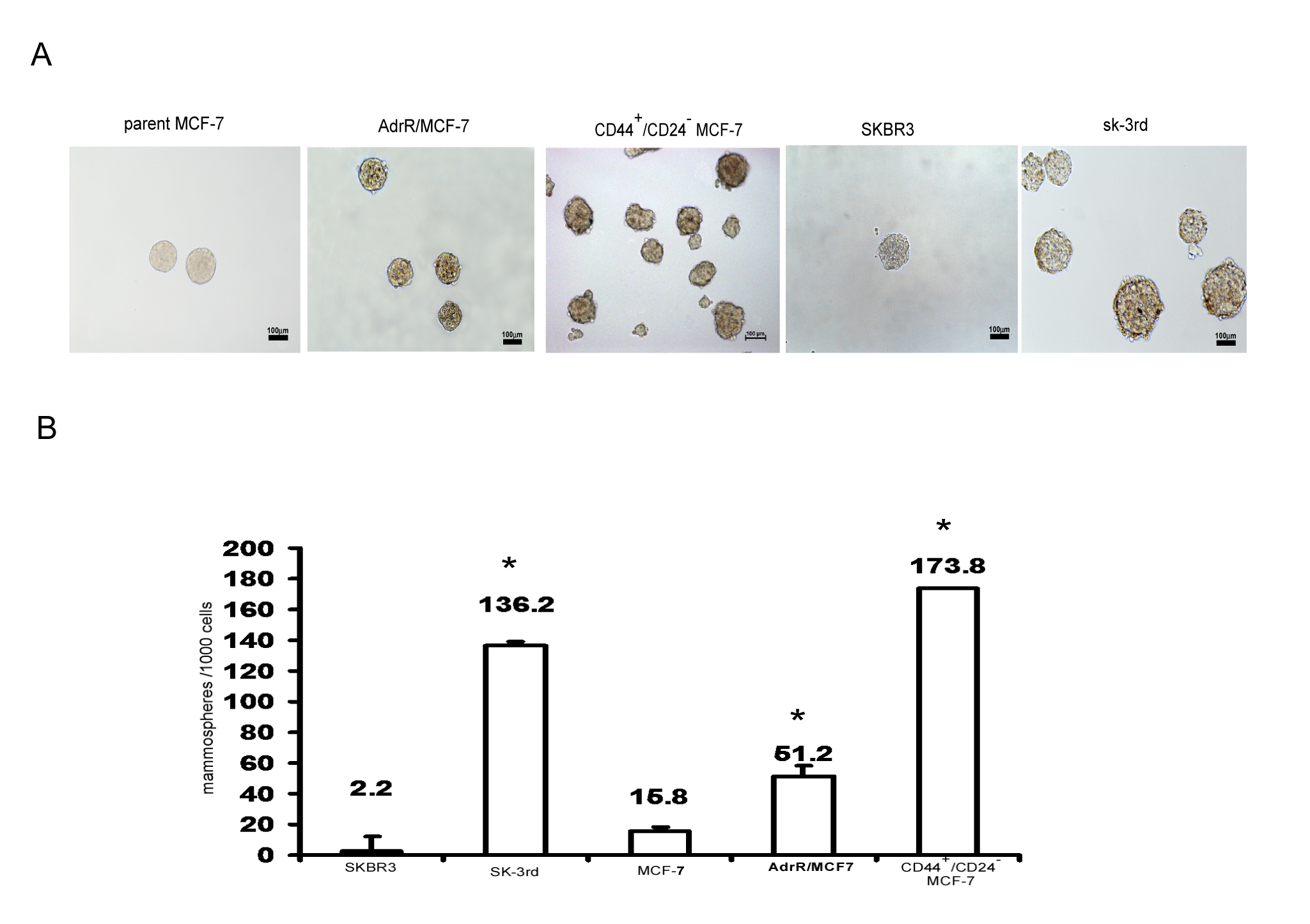

Supplement: Figure S2 — Mammospheres forming in chemotherapy sensitive and resistant breast cancer cells. (A) Representative images of mammospheres for parental MCF-7, AdrR/MCF-7, CD44+/CD24− MCF-7, SKBR3 and SK-3rd. (B) The percentage of mammospheres formed in CD44+/CD24− MCF-7, AdrR/MCF-7 and sk-3rd cell lines was significantly higher than parental MCF-7 line and SKBR3 line (*p<0.001,mean±SD). (TIF) [file pone.0015630.s002.tif]

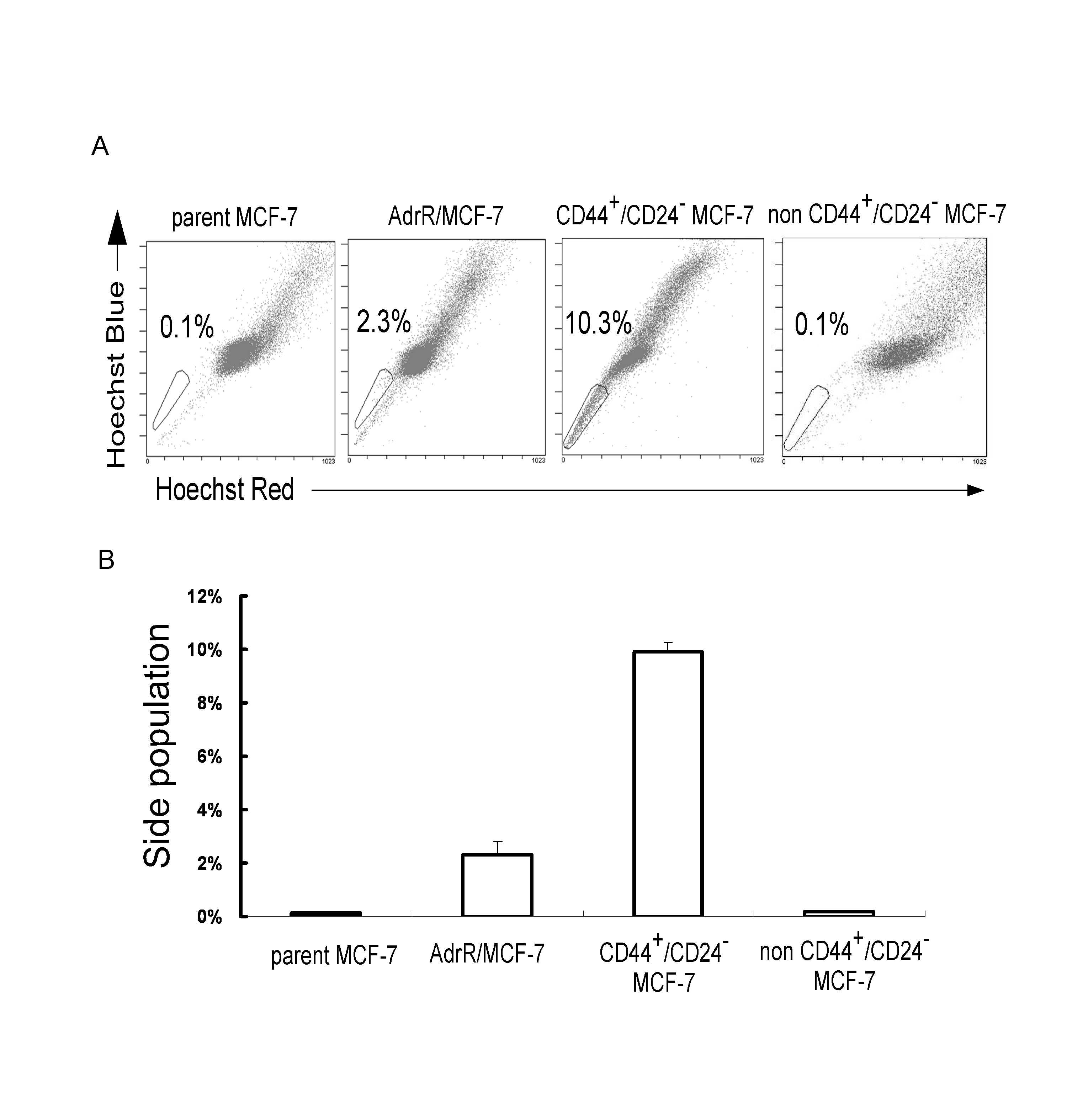

Supplement: Figure S3 — High proportion of side-population (SP) in CD44+/CD24− cells. (A) Representative images of SP determined by FACS analysis. (B) Compared with non-CD44+CD24− MCF-7, parental MCF-7 and AdrR/MCF-7, CD44+CD24− MCF-7 was more sensitive to adriamycin (*p<0.01, **p<0.001, mean±SD). Results were from 3 independent experiments. (TIF) [file pone.0015630.s003.tif]

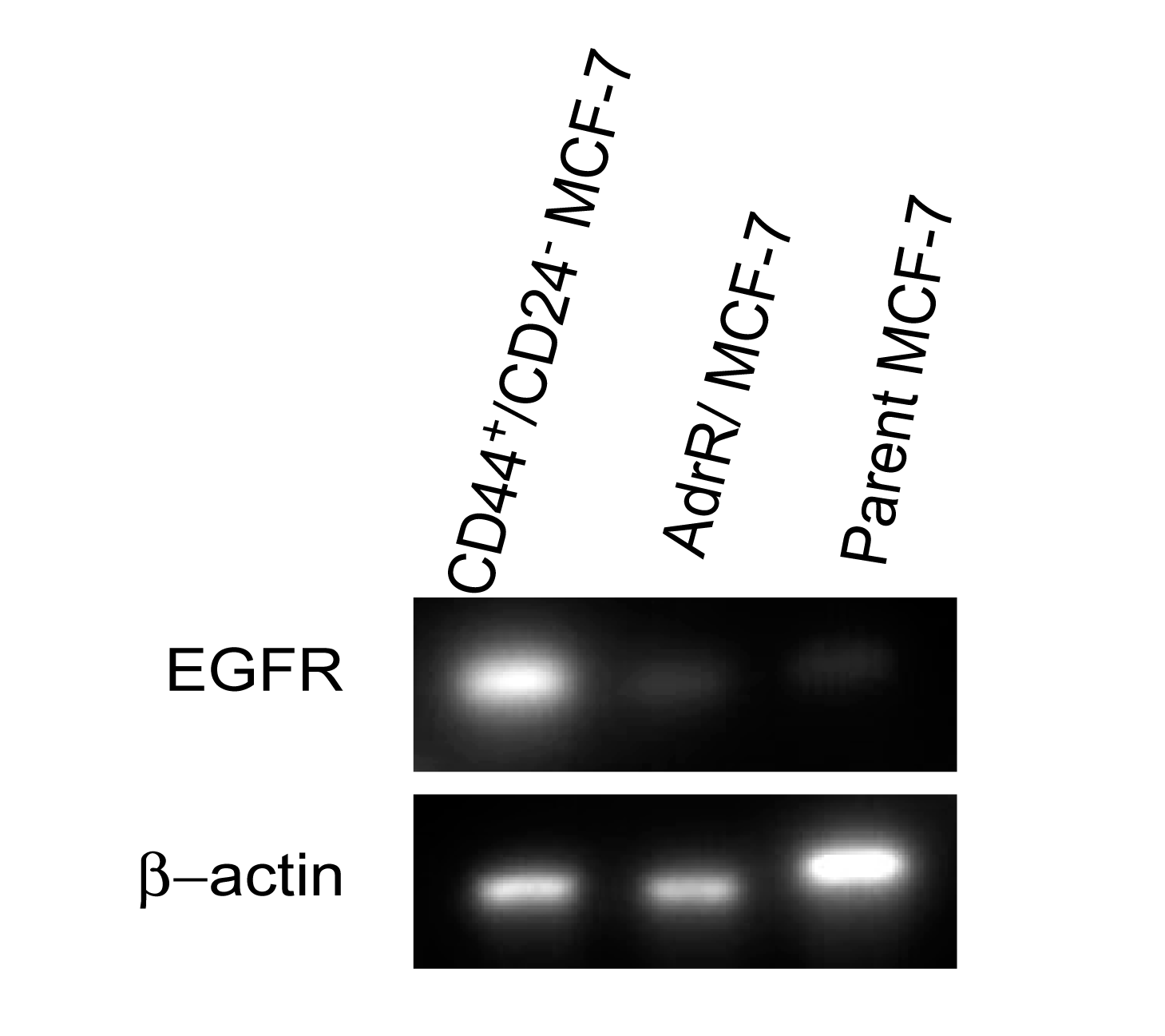

Supplement: Figure S4 — Expression of EGFR in breast cancer cell lines. In comparison to the parental MCF7 cells and AdrR/MCF7 cells, CD44+/CD24− MCF-7 cells more constitutively expressed EGFR tested by RT-PCR. This experiment was repeated 3 times independently. (TIF) [file pone.0015630.s004.tif]

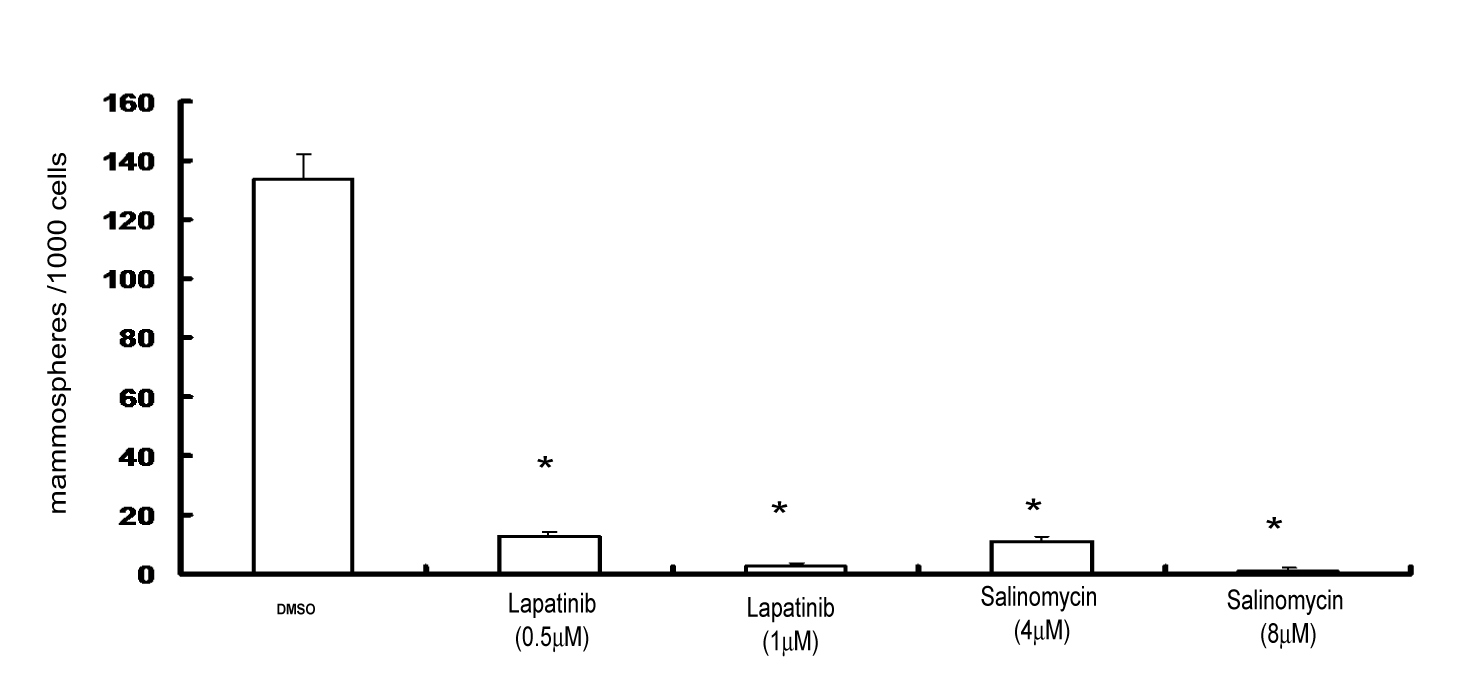

Supplement: Figure S5 — Salinomycin or lapatinib significantly reduced mammosphere formation of sk-3rd cells by approximately 10–100 fold relative to DMSO control (p<0.001). This experiment was repeated 3 times independently. (TIF) [file pone.0015630.s005.tif]

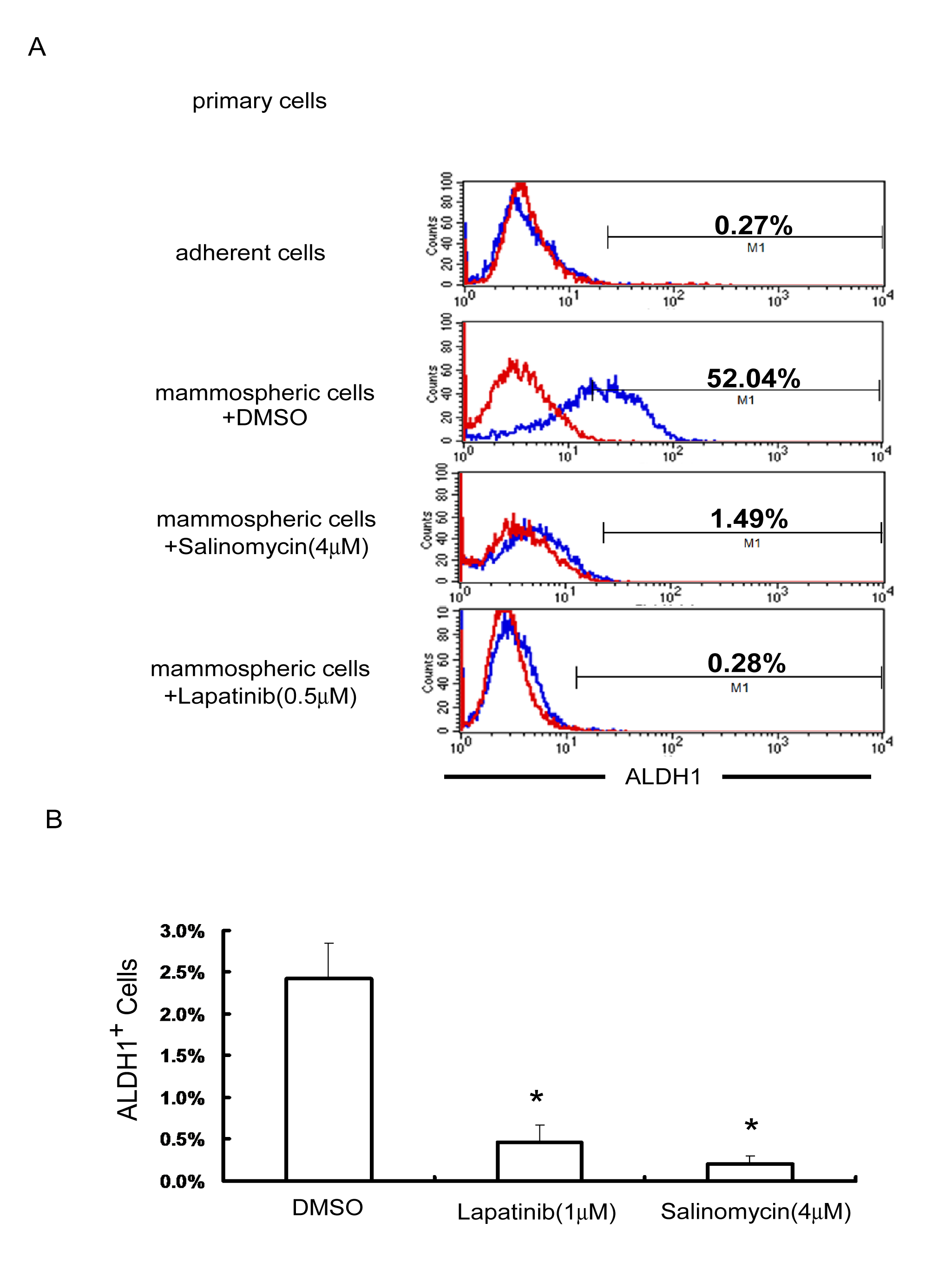

Supplement: Figure S6 — Treatment with salinomycin or lapatinib reduced the percentage of ALDH1+ in sk-3rd cells and primary cells by 10–50 folds. (TIF) [file pone.0015630.s006.tif]

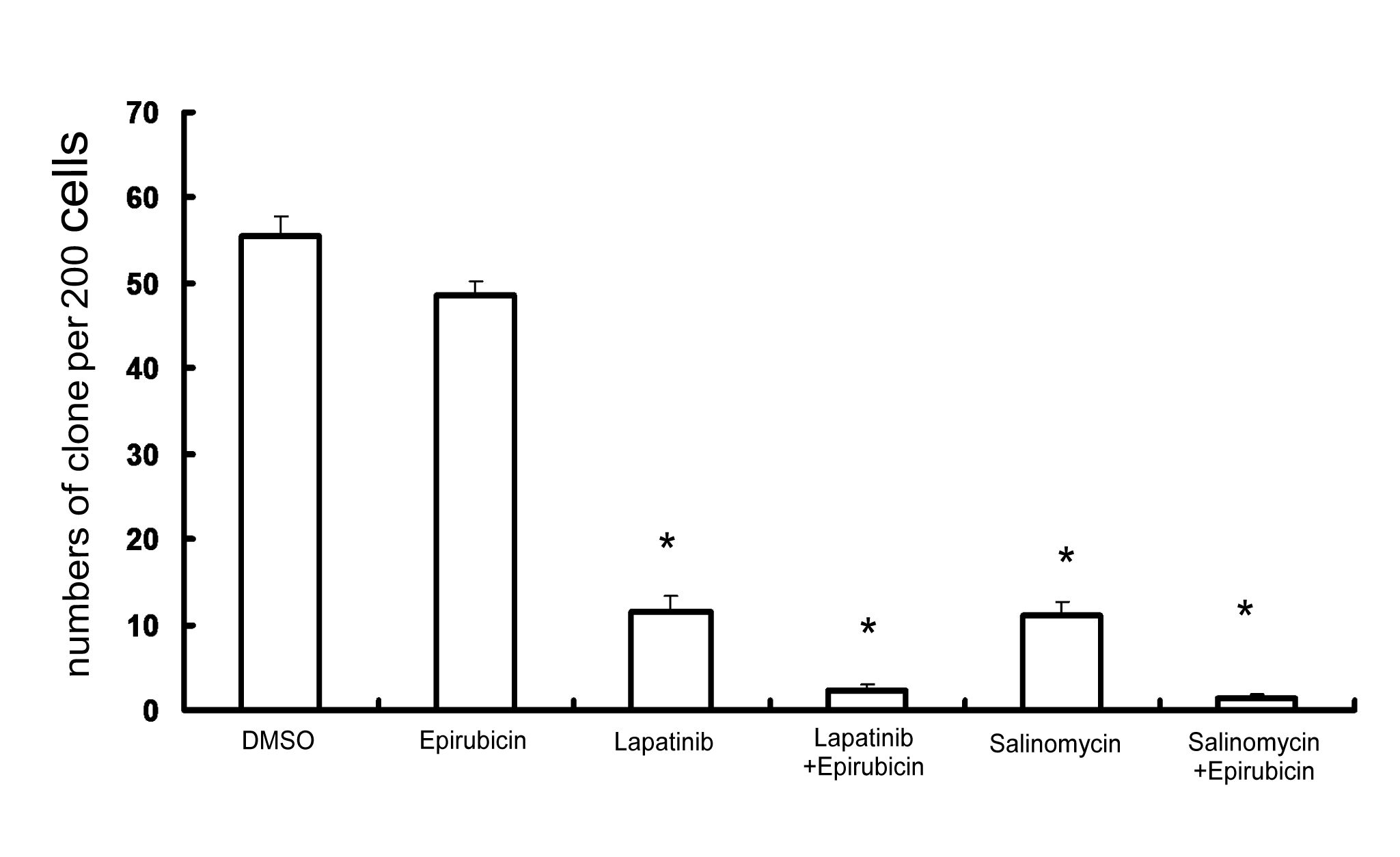

Supplement: Figure S7 — Clone forming assay from 3 independent experiments showed that epirubicin combined with salinomycin and lapatinib reduced the cloning efficiency of SK-3rd cells compared with epirubicin alone (p<0.001). (TIF) [file pone.0015630.s007.tif]
