# Supplementary material for: Markers of Tumor-Initiating Cells Predict Chemoresistance in Breast Cancer
Source: PLoS One. 2010 Dec 20;5(12):e15630. doi: 10.1371/journal.pone.0015630 (PMC3004932; doi:10.1371/journal.pone.0015630)
Supplement: Table S1 — (DOC) [file pone.0015630.s008.doc]

Table S1

The treatments that patients received in high and low ALDH1 expression are comparable

Therapy ALDH1 ALDH1 P value

≤20% ＞20%

Endocrine therapy^※^

AI 39(34.51%) 8(30.77%) 0.876

Tamoxifen 74(65.49%) 18(69.23%)

Chemotherapy﹟

CEF 98(84.48%) 10(45.45%) 0.124

Taxol 18(15.52%) 12(54.55%)

- Analyzed in 139 ER- positive patients.﹟All the patients underwent pre-operative neoadjuvant chemotherapy

with 2 -6 cycles of FEC regimen (5-Fluorouracil 500mg/m^2^, Epirubucin 90mg/m^2^ and Cyclophosphamide 500mg/m^2^). According to the clinical response，continously adjuvant FEC or transformed Taxotere was selectively administred in the patients with PR/cCR or PD/SD after surgery.16 patients received other chemotrapy were not analyzed .
