# Supplementary material for: Markers of Tumor-Initiating Cells Predict Chemoresistance in Breast Cancer
Source: PLoS One. 2010 Dec 20;5(12):e15630. doi: 10.1371/journal.pone.0015630 (PMC3004932; doi:10.1371/journal.pone.0015630)
Supplement: Table S2 — (DOC) [file pone.0015630.s009.doc]

Table S2

Survival table for the number of events in 192 stage IIB / III breast cancer patients

Time(months) ALDH1≤20%（154） ALDH1＞20%（38）

Metastasis(33) death( 28 ) Metastasis (21 ) death( 15 )

20 1

22 1

24 7 3 5

26 1 2

27 1 1

28 3 2 2

30 5 4 3

31 1

32 1

34 2

36 4 4 3

38 3 1 1

40 1 3 1 3

42 2 3 1 1

43 1

45 1 1

48 3 1 2

50 1 3 2

52 1 1

54 1

56 1

60 1

62 1
