# Supplementary material for: Markers of Tumor-Initiating Cells Predict Chemoresistance in Breast Cancer
Source: PLoS One. 2010 Dec 20;5(12):e15630. doi: 10.1371/journal.pone.0015630 (PMC3004932; doi:10.1371/journal.pone.0015630)
Supplement: Table S3 — (DOC) [file pone.0015630.s010.doc]

Table S3

Cox multivariate analysis of various prognostic variables in patients with breast cancer

| **Variables** | **Subgroup** | **P** | **Relative risk** | **95% confidence interval** |
| --- | --- | --- | --- | --- |
| Tumor size | ≤2cm | 0.003 | 1.354 | 1.106-1.658 |
|  | ＞2cm |  |  |  |
| ER | Positive | 0.224 | 0.650 | 0.315-1.341 |
|  | Negative |  |  |  |
| HER2 | Positive | 0.006 | 2.298 | 1.271-4.153 |
|  | Negative |  |  |  |
| Clinical Stage | II | 0.089 | 0.525 | 0.250-1.103 |
|  | III |  |  |  |
| Grade | Ⅰ | 0.737 | 1.086 | 0.671-1.760 |
|  | II & III |  |  |  |
| Node Metastasis | N_1_ | 0.003 | 1.354 | 1.106-1.658 |
|  | N_2_-N_3_ |  |  |  |
| PCNA | ≤25% | 0.734 | 0.874 | 0.402-1.901 |
|  | ＞25% |  |  |  |
| AI |  | 0.332 | 1.386 | 0.717-2.680 |
| ALDH1 | ≤20% | 0.013 | 2.129 | 1.174-3.863 |
|  | ＞20% |  |  |  |

ALDH1= aldehyde dehydrogenase 1; AI=apoptotic index, percentage of apoptotic cancer cells.
